# The reconsolidation using rewind study (RETURN): trial protocol

**DOI:** 10.1080/20008198.2020.1844439

**Published:** 2021-01-28

**Authors:** Laurence Astill Wright, Kali Barawi, Natalie Simon, Catrin Lewis, David Muss, Neil P. Roberts, Neil J Kitchiner, Jonathan I Bisson

**Affiliations:** a Division of Psychological Medicine and Clinical Neurosciences, Cardiff University School of Medicine, Cardiff, UK; b International Association for Rewind Trauma Therapy, UK; c Directorate of Psychology and Psychological Therapies, Cardiff & Vale University Health Board, Cardiff, UK

**Keywords:** PTSD, RCT, Rewind Technique, Rewind Therapy, protocol, TEPT, ECA, Técnica de Rebobinado, Terapia de Rebobinado, Protocolo, PTSD, RCT, Rewind技术, Rewind疗法方案

## Abstract

**Background:** An increasing body of research highlights reconsolidation-based therapies as emerging treatments for post-traumatic stress disorder (PTSD). The Rewind Technique is a non-pharmacological reconsolidation-based therapy with promising early results, which now requires evaluation through an RCT.

**Objectives:** This is a preliminary efficacy RCT to determine if the Rewind Technique is likely to be a good candidate to test against usual care in a future pragmatic efficacy RCT.

**Methods:** 40 participants will be randomised to receive either the Rewind Technique immediately, or after an 8 week wait. The primary outcome will be PTSD symptom severity as measured by the Clinician-Administered PTSD Scale for DSM5 (CAPS-5) at 8 and 16 weeks post-randomisation. Secondary outcome measures include the PTSD Checklist (PCL-5), International Trauma Questionnaire (ITQ), Patient Health Questionnaire (PHQ-9), the General Anxiety Disorder-7 (GAD-7), Insomnia Severity Index, the Euro-Qol-5D (EQ5D-5 L), the prominence of re-experiencing specific symptoms (CAPS-5) and an intervention acceptability questionnaire to measure tolerability of the intervention.

**Conclusions:** This study will be the first RCT to assess the Rewind Technique. Using a cross-over methodology we hope to rigorously assess the efficacy and tolerability of Rewind using pragmatic inclusion criteria. Potential challenges include participant recruitment and retention.

**Trial registration:** ISRCTN91345822

## HIGHLIGHTS

- This study will be the first RCT to rigorously assess the efficacy and tolerability of the Rewind Technique.

## Abstract (es)

**Antecedentes:** Un creciente cuerpo de investigación destaca las terapias basadas en la reconsolidación como tratamientos emergentes para el trastorno de estrés postraumático (TEPT). La Técnica de Rebobinado es una terapia no farmacológica basada en la reconsolidación con resultados tempranos prometedores, que ahora requiere evaluación a través de un ECA.

**Objetivos:** Este es un ECA preliminar de eficacia para determinar si es probable que la técnica de rebobinado sea una candidata adecuada para probar en comparación con el cuidado habitual en un futuro ECA de eficacia pragmática.

**Método:** 40 participantes serán asignados al azar para recibir la técnica de rebobinado inmediatamente o después de una espera de 8 semanas. El resultado primario será la gravedad de los síntomas del TEPT según lo medido por la Escala de TEPT administrada por el médico para DSM5 (CAPS-5 en su sigla en inglés) a las 8 y 16 semanas posteriores a la aleatorización. Las medidas de resultados secundarios incluyen la Lista de Verificación de TEPT (PCL-5 en su sigla en inglés), el Cuestionario Internacional de Trauma (ITQ en su sigla en inglés), el Cuestionario de Salud del Paciente (PHQ-9 en su sigla en inglés), el Trastorno de Ansiedad General-7 (GAD-7 en su sigla en inglés), el Índice de Gravedad del Insomnio, el Euro-Qol- 5D (EQ5D-5L en su sigla en inglés), la prominencia de re-experimentar los síntomas específicos (CAPS-5) y un cuestionario de aceptabilidad de la intervención para medir la tolerabilidad de la intervención.

**Conclusiones:** Este estudio será el primer ECA para evaluar la Técnica de Rebobinado. Utilizando una metodología cruzada, esperamos evaluar rigurosamente la eficacia y tolerabilidad del Rebobinado utilizando criterios de inclusión pragmáticos. Los desafíos potenciales incluyen el reclutamiento y la retención de los participantes.

## Abstract (zh)

**背景:** 越来越多的研究强调, 基于 (记忆) 再固化是创伤后应激障碍 (PTSD) 的新兴疗法。Rewind 技术 (Rewind Technique) 是一种非药物的基于再固化的疗法, 具有可喜的早期结果, 现在需要通过RCT进行评估。

**目的:** 本初步效能RCT的结果用于确定Rewind技术是否可能在将来的实用效能RCT中作为潜在选择方案针对常规护理进行对比评估。

**方法:** 40名参与者将被随机分配为立即接受Rewind 技术或等待8周。主要结果将是PTSD症状严重程度, 在随机分配后8周和16周由DSM-5的临床PTSD量表 (CAPS-5) 测得。次要结果指标包括PTSD检查表 (PCL-5), 国际创伤问卷 (ITQ), 患者健康问卷 (PHQ-9), 广泛性焦虑障碍量表7 (GAD-7), 失眠严重度指数, Euro-Qol- 5D (EQ5D-5L), 出现特定再体验症状的显著性 (CAPS-5) 和测量干预耐受度的干预接受度问卷。

**结论:** 本研究将是第一个评估Rewind 技术的RCT。通过使用交叉设计 (cross-over) 方法, 我们希望用实用的纳入标准来严格评估Rewind的功效和耐受性。潜在的挑战包括参与者的招募和保留。

## 1. Background and rationale

Post-traumatic stress disorder (PTSD) is a common mental illness occurring after a traumatic event, with symptoms of re-experiencing, hyper-arousal, negative alterations in cognition and mood, and avoidance. It has been estimated that between 1.1% and 2.9% of EU adults suffer from PTSD (Wittchen et al., 2011) and the prevalence of PTSD in high-risk populations such as those exposed to certain types of interpersonal trauma, mass conflict and displacement are estimated to be much higher (Kessler et al., 2017; Steel et al., 2009). PTSD is associated with substantial physical and psychiatric co-morbidity, for example type 2 diabetes, ischaemic heart disease, somatic symptom disorder, substance abuse and suicide (Roberts et al., 2015) and research suggests that current psychological and pharmacological treatments remain ineffective in a substantial proportion of those with PTSD (Olff et al., 2019, Lewis et al. 2020). Trauma-focused psychological therapies in the form of individual trauma-focused cognitive behavioural therapy (TFCBT) and eye movement desensitisation and reprocessing (EMDR) are currently the most effective treatments for PTSD symptoms with pharmacological approaches effective but to a lesser degree (National Institute for Health and Care Excellence (NICE), 2018, Bisson et al. 2019). Sadly, many people with PTSD remain symptomatic, despite evidence-based treatment, partially due to high drop out rates in trauma-focused therapies, which appear particularly difficult to tolerate (Lewis et al. 2020). There is an urgent need to develop more effective treatments (Olff et al., 2019), which are shorter in duration and less emotionally distressing (Lewis et al. 2020).

The positive outcomes of recent studies of novel treatments have raised interest in the mechanism and study of memory reconsolidation and further work in this area is merited to establish the exact mechanism of action (Brunet et al., 2018; Gray, Budden-Potts, & Bourke, 2017). As memory reconsolidation has been specifically targeted by some novel treatments (Gray et al., 2017) which have many similarities to the Rewind technique (the psychological intervention assessed here) we will consider reconsolidation theory in detail below.

Memory consolidation is the process where short-term memories are converted into long-term ones, while in memory reconsolidation long-term memories are reactivated, changed and then updated. Memories of stressful and fearful events have a large degree of salience and so are laid down particularly concretely within the biology of the brain, giving rise to PTSD symptoms. Fearful memories are made in periods of high emotional arousal and the formation of these is strengthened by the stress hormones that contribute to initial memory consolidation. Cortisol (De Quervain et al., 2003) and adrenaline are released during periods of significant stress, and these regulate memory consolidation by causing noradrenergic excitement of the basolateral amygdala (McGaugh, 2015; Roozendaal & Hermans, 2017; Schwabe & Bolam, 2017). While this is not exclusive to fearful memories, and heightened memory consolidation occurs in a variety of emotional experiences, it is argued that disrupted memory consolidation plays a key role in the development and maintenance of the characteristic symptoms of PTSD, such as hyperarousal and re-experiencing (Pitman, 2019).

Despite this, emotional memory is highly malleable (Pitman, 2019) and a number of medications are believed to be able to interrupt reconsolidation, with the potential therapeutic effect of decreasing PTSD symptoms. Animal research has shown that neuroplasticity is not only required for the creation of new emotional memories but also for the removal of fear memories (Nader, Schafe, & Le Doux, 2000). The neuroplasticity of memory consolidation requires protein synthesis, a process which can be interrupted to prevent learning consolidation and create amnesia of a fearful event (Nader et al., 2000). Furthermore, when already consolidated fear memories are remobilised, new protein synthesis can facilitate reconsolidation, providing an opportunity to reconsolidate memories and decrease their emotional salience (Nader et al., 2000). Once a memory becomes reactivated and the memory is returned to a mobilised state similar to pre-consolidation, the protein synthesis antagonist anisomycin can be given to block this reconsolidation (Pitman, 2019). While anisomycin’s toxicity prohibits its use as a therapy in humans, its action provides understanding of how the mobilisation of fear memories may ameliorate PTSD symptoms (Suris, Smith, Powell, & North, 2014). While exposure-based therapies may inhibit the body’s reaction to the traumatic memory, or impair its retrieval (de Quervain, Schwabe, & Roozendaal, 2017), memory reconsolidation treatments aim to mobilise traumatic memories to remove these pathognomonic symptoms entirely (de Quervain et al., 2017; Pitman, 2019).

It may, however, not be necessary to use pharmacological approaches to augment/facilitate reconsolidation. Non-pharmacological approaches may also be effective and would likely be preferred and better tolerated by many people with PTSD (Feeny, Zoellner, & Shoshana, 2009), and avoid the contraindications for some pharmacotherapies (e.g. asthma with propranolol). Reconsolidation of Traumatic Memories (RTM) is a non-pharmacological approach that aims to treat PTSD through memory reconsolidation over three sessions (Gray, 2011). The proven mechanism of RTM remains unclear, however, and additional factors, such as the reactivation and redistribution of emotional memories in the neocortex during sleep, may also play a role (Schafer et al., 2020). RTM (Gray, 2011) has RCT evidence of effect leading to it being recommended as a treatment with emerging evidence by the ISTSS Treatment Guidelines for PTSD and for military populations (Bisson et al, 2019; Kitchiner, Lewis, Roberts, & Bisson, 2019). RTM is a development of the Rewind Technique, another non-pharmacological approach, with many similarities in its delivery to RTM, but with an evidence base that is currently reliant on non-randomised trials (Adams & Allan, 2018; Utuza,, Joseph, & Muss, 2012). RTM and Rewind both employ ‘rewinding’ techniques (where the participant plays their trauma memory in reverse) using different intervention protocols.

The Rewind Technique and RTM seek to briefly mobilise traumatic memories, before exploring dissociative experiences surrounding the trauma which are proposed to modify the memory, reconsolidating it so that it can be remembered without evoking PTSD symptomatology (Tylee, Gray, Glatt, & Bourke, 2017). The brief memory mobilisation stimulus is thought to be too brief to produce effects via improving the extinction of the traumatic memory (Merlo, Milton, Goozée, Theobald, & Everitt, 2014), and follow up suggests that the rapid reacquisition or reinstatement of traumatic memories, that might be expected if the underlying mechanism was one of memory extinction, does not occur (Gray et al., 2017).

Some reconsolidation approaches have yielded negative results (Bos, Beckers, & Kindt, 2014; Wood et al., 2015) highlighting the specific limitations which exist around the ability of a therapy to destabilise and modify a traumatic memory. These factors, such as the length of the memory reactivation procedure and the age and context of the traumatic memory, are known as boundary conditions (Treanor, Brown, Rissman, & Craske, 2017) and have complicated the process of translation from experimental into clinical settings. Promising preliminary evidence, however (Adams & Allan, 2018; Utuza, et al., 2012), suggests that if reconsolidation is the mechanism of action for the Rewind technique, that these conditions may have been met.

While trauma-focused psychotherapies (TFCBT, EMDR) have medium to large effect sizes, the therapy requires large amounts of therapist time (Lewis et al. 2020). We expect that Rewind will have a similar effect size, but be more time and cost efficient, delivering a trauma-focused intervention in just a few sessions. Furthermore, as Rewind is relatively simple to deliver it has potential to be delivered by lower intensity therapists and therefore is more easily scalable. Furthermore, evidence of multiple effective therapies also provides treatment choice for people with PTSD, e.g. some individuals may prefer Rewind as it does not require trauma disclosure. Considering this, Rewind may be most suitable as a first-line intervention as part of a stepped care approach.

Given the promise of non-pharmacological approaches to the treatment of PTSD through the mechanism of memory reconsolidation, we aim to undertake a preliminary efficacy RCT to determine if the Rewind Technique is likely to be a good candidate to test against usual care in a future pragmatic efficacy RCT. Our objectives are:

1. To investigate the effect sizes of the Rewind Technique at reducing PTSD symptoms in people with PTSD.

2. To establish whether any symptom improvement is maintained over 16 week follow up.

3. To investigate the impact of the Rewind Technique on symptoms of depression, anxiety and insomnia.

4. To investigate if an efficacy RCT is feasible and indicated.

5. To assess if the Rewind Technique is acceptable to participants with PTSD as measured by a qualitative questionnaire.

## 2. Methods

### 2.1. Study design

The study will be a two-armed, phase 2 exploratory RCT to assess the preliminary efficacy, adherence, feasibility and factors affecting outcome of the Rewind Technique versus a waitlist control group (Table 1).

**Table 1.** Gantt chart illustrating study timeline

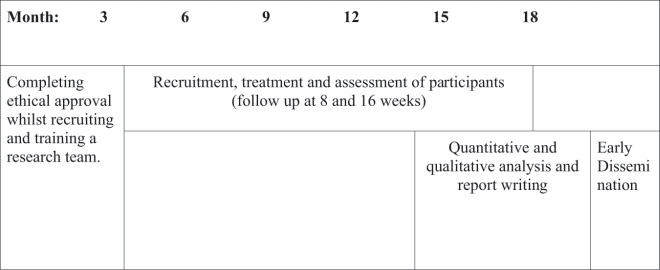

### 2.2. Setting

Participants will be people with PTSD living in South Wales. Recruitment will primarily occur through University Health Boards in South Wales using approaches that have proved successful in other RCTs run by Cardiff University’s Traumatic Stress Research Group (Lewis et al., 2017; Nollett et al., 2018).

### 2.3. Sample size

While a definitive power calculation is not necessary for a phase 2 exploratory trial, it is nonetheless important to ensure our sample size is appropriate and adequate. We conservatively based this power calculation on the broad range of effect sizes reported for trauma-focused psychological therapies for PTSD. While it is difficult to compare the wide range of effect sizes reported in heterogeneous trials, as meta-analyses demonstrate (Lewis et al. 2020, Mavranezouli et al., 2020), we considered an effect size of 1.45 highly clinically relevant.

Using the Lehr method to give a sample size with 80% power, a 5% significance level and an effect size of 1.5, 15 participants are required in each arm of the trial, with a total number of 30. Estimating a conservative 25% attrition rate, informed by recent similar research (Lewis et al. 2020), we will include 20 participants in each arm of the trial, giving 40 participants in total.

### 2.4. Inclusion/exclusion criteria

In order to determine the likely efficacy of the Rewind Technique for people with PTSD presenting to the UK’s National Health Service (NHS) we will adopt a pragmatic approach and employ broad eligibility criteria.

Inclusion criteria will be: adults aged 18 or over who are fluent in English, able to provide informed consent, and who meet DSM-5 criteria for PTSD secondary to a single traumatic event.

Exclusion criteria will be: current psychotic or bipolar disorder, traumatic brain injury, substance dependence, acute suicidal ideation, learning disability, previous or current receipt of an adequate trial of trauma-focused psychological treatment for PTSD, change to the type or dosage of psychotropic medication within one month of baseline assessment. The presence of other mental disorders will be allowed as long as PTSD is the primary condition, and pretreatment comorbidity will be assessed using clinical presentation and self-report.

### 2.5. Recruitment and consent

Potential study participants who are thought likely to fulfil the inclusion criteria for the trial will be asked by a clinician involved in their care if they are willing for their details to be passed on to the research team. Participants will also be recruited via the National Centre for Mental Health (NCMH), a Welsh government-funded research centre (NHS Health Research Authority, 2016), which recruits through primary and secondary mental health services as well as through a variety of media outlets and online advertising strategies. The research team will contact potential study participants and provide them with an information leaflet about the research. After 24 hours they will be asked if they would like any further information or have any queries they would like answered. If potential participants wish to proceed, arrangements will be made to enter them into the study through recruitment screening.

Participants will provide fully informed consent to take part in the study before their initial assessment. The individual taking consent will check that the participant has read and understood the information sheet provided and answer any participant questions that arise. They will check the participant’s understanding that their participation is voluntary and that they are free to withdraw (on a variety of levels) at any time without giving any reason (without their medical care or legal rights being affected), and ask if the participant agrees to their GP being informed of their participation in the study. Data will be handled with full compliance to the General Data Protection Regulations 2016.

### 2.6. Outcome measures

A trained member of the research team, blind to randomization, will conduct all clinical assessments. It is not possible for therapists or participants to be blind to treatment allocation, but participants will be asked not to discuss their allocation with their assessor. An initial telephone screen will confirm that the participant meets the inclusion criteria. To ensure they consistently meet the inclusion criteria, participants will be asked to monitor their symptoms for two weeks, at which point we will take informed consent, collect baseline demographic data, outcome measurements, reassess eligibility, and then randomise eligible individuals to one of two groups using a computer programme: the Rewind Technique immediately or a waitlist for eight weeks followed by the Rewind Technique. We will monitor change in PTSD, depressive and anxiety symptoms using the PTSD Checklist (PCL-5), Patient Health Questionnaire (PHQ-9), and General Anxiety Disorder-7 (GAD-7) at the start of each treatment session. Follow-up and repeated collection of outcome data will occur 8 and 16 weeks post-randomisation.

The primary outcome of PTSD symptom severity, and PTSD diagnosis will be measured by trained assessors using the Clinician Administered PTSD Scale for DSM-5 (CAPS-5) (Weathers et al., 2018). The CAPS-5 is widely referred to as the gold standard in PTSD assessment, demonstrating high internal consistency (α =.88) and strong test-retest reliability (к = .83) (Weathers et al., 2018). Measuring DSM-5 PTSD allows comparability with previous psychological therapy RCTs.

Secondary outcomes, collected at 8 and 16 weeks will include:

The PTSD Checklist (PCL-5), a validated and widely used self-report measure for DSM-5 PTSD symptoms (Ruggiero, Del Ben, Scotti, & Rabalais, 2003).

The International Trauma Questionnaire (ITQ), a validated self-report measure for ICD-11 PTSD and complex PTSD (Cloitre et al., 2018).

The Patient Health Questionnaire-9 (PHQ-9), a validated and widely used self-report measure for assessing DSM-5 depressive symptoms (Kroenke, Spitzer, & Williams, 2001).

The Generalised Anxiety Disorder Assessment-7 (GAD-7), a validated and widely used self-report measure for assessing symptoms of generalised anxiety disorder (Spitzer, Kroenke, Williams, & Lowe, 2006).

The Insomnia Severity Index (ISI), a validated and widely used self-report measure for symptoms of insomnia, such as prolonged sleep latency and effects on functioning, over the past month, using a five-point Likert scale (Morin, Belleville, Bélanger, & Ivers, 2011).

The five-level EQ-5D (EQ5D-5 L), a validated and widely used self-report measure for health-related quality of life. It is recognised by NICE as such and is frequently used in health economic analyses (EQ-5D, 2017).

An intervention acceptability questionnaire to gauge acceptability and feasibility of the intervention, as perceived by the participant, will be completed at the end of the final session and returned to a member of the research team in a sealed envelope. Feasibility will be partly assessed through dropout numbers.

The prominence of re-experiencing symptoms will be assessed through a score of ≥3 on CAPS-5 items B1, B2 or B3).

### 2.7. Intervention

*The Rewind Technique –* This will comprise up to three 60-minute sessions following a protocol developed by David Muss (Muss, 1991) which has been modified by David Muss and the research team following feedback from therapists. The intervention will be administered by experienced and trained psychological therapists under the supervision of David Muss and the Cardiff and Vale University Health Board Traumatic Stress Service. The participant will be introduced to the technique and the theory behind it before being asked to imagine he/she is in a cinema watching a film of her/his traumatic event as if it had been captured on CCTV. Rather than the film start at the trauma itself the person with PTSD is told the film starts just before the traumatic event took place and is then followed by the regular intrusive recall which includes all the images, sounds and smells plus (if this is part of the regular recall) what could have happened next but didn’t. Once the recall ends, the sufferer is (metaphorically) invited to enter the screen and at that point the film is rewound at speed back to the exact starting point (where all was well before the trauma). The forward part of the process of recalling the trauma should not take longer than 2 minutes, the rewind part about 10 seconds. This usually requires the person with PTSD to practise the technique a few times before feeling confident it is being undertaken as intended.

*Wait list* – The wait list group will receive no intervention for 8 weeks after randomisation, at which point they will then receive the Rewind Technique in the same manner as the immediate treatment group.

Participants will be randomised to receive either the Rewind Technique or wait list using a 1:1 ratio.

### 2.8. Study timeline

Once ethical approval is granted, the study is planned to take place over 18 months, with data collection completed within 12 months, and the remaining time being used for data analysis, report preparation and early dissemination as illustrated by the Gantt chart below.

### 2.9. Planned analyses

We will conduct intention-to-treat analyses on all randomised participants. We will compare the means of continuous data using ANCOVA with baseline outcomes (such as CAPS-5 score) as co-variates. Risk ratios will be calculated for categorical data. The factors associated with reduction in symptom severity scores will be analysed using regression (baseline CAPS-5 score, age, prominence of re-experiencing symptoms, depressive symptoms, educational attainment and employment status), with all analyses performed at the end of data collection using SPSS. The dataset will be cleaned and then ‘locked’ before the randomisation codes are revealed and the final analyses undertaken. This will ensure those conducting the analysis are blind to condition.

### 2.10. Dissemination of findings

Research findings will initially be communicated in lay terminology to study participants, with a research report and an end of study meeting. Participants and the public will be involved in the dissemination of the research findings in collaboration with the Cardiff University Traumatic Stress Research Group’s Patient and Public Involvement Group, consisting of five members of the public with lived experience of PTSD. We will present the work at academic conferences and facilitating workshops to inform future work in this area.

We will publicise both the running of the trial and its results through the NCMH communications team. Furthermore, there will be a study-specific webpage, under the NCMH website, which will congregate all the information around the trial, including lay and academic summaries and press releases.

### 2.11. Data management

Personal data will be stored on University computers, with personal data kept separately to anonymised clinical data. Only the direct care and research team will have access to participants’ personal data during the research study.

## 3. Discussion

This phase 2 exploratory trial will assess the preliminary efficacy, acceptability and feasibility of the Rewind Technique to determine if an effectiveness RCT is indicated. If a phase 3 RCT is warranted, investigating possible effect sizes of the intervention in this trial will enable us to conduct an appropriate power calculation. This will be the first RCT evaluating Rewind, with previous non-randomised work highlighting the need for randomised trials, particularly those with long term follow up (Adams & Allan, 2018). Previous pilot work has assessed outcomes 2 weeks post-intervention, while we will follow-up participants 8 and 16 weeks post-randomisation. While RTM has been investigated in randomised trials and shares many characteristics with Rewind (Gray, 2011), it is fundamentally a different intervention and both require evaluation separately.

We will also improve on previous research by using the ‘gold standard’ of PTSD assessment, the CAPS-5, to measure our primary outcome. Previous pilot work shows the intervention to be well tolerated (Adams & Allan, 2018), and our involvement of patient and public representatives in the production of our participant information sheets, consent forms and trial protocol aims to improve the acceptability of recruitment and the intervention itself. Furthermore, considering the urgent need for new treatments for PTSD, and the current prevalence of Rewind already as a therapy in the UK (IARTT (International Association for Rewind Trauma Therapy), 2019), new evidence is clearly needed to assess the long-term effectiveness of Rewind on symptoms of PTSD, anxiety, depression and insomnia. While this trial is not powered to definitively assess effectiveness, it will provide the highest quality of evidence available for the Rewind technique.

This study with relatively narrow inclusion criteria will recruit new patients through local NHS health boards. Including any individual with a PTSD diagnosis from a singular traumatic event that does not meet the exclusion criteria will create a heterogeneous cohort, with a variety of different experiences from a wide possibility of traumas, representative of people with PTSD treated by the NHS. While this is crucial to create the quality of evidence required by NHS commissioners, there are numerous challenges associated with this. PTSD naturally includes those with high levels of avoidance and anxiety, and this may make recruitment and retention within the trial difficult. This is, however, a challenge that all trauma-focused psychological therapies must cope with, and the Rewind technique may be preferable to participants as it requires fewer sessions than current best evidenced techniques, such as trauma-focused CBT. Rewind potentially offers a trauma-focused intervention without lengthy periods of intense imaginal exposure or disclosure of trauma. This may be more tolerable to participants, requiring less clinical contact than traditional therapies and possibly decreasing the risk of secondary traumatisation and compassion fatigue from therapists.

High levels of distress and avoidance may also cause some participants to struggle to appropriately reactivate the memory of their trauma (although there is clinical risk management procedure for adverse effects of the therapy), which we know is fundamental to then reconsolidate memory and thus reduce PTSD symptoms (Kindt & Van Emmerik, 2016). Incorporating three Rewind sessions within the intervention may help to increase the likelihood that participants engage with this crucial memory reactivation. Furthermore, this trial will use therapists trained in the Rewind technique, with the therapy following a semi-structured protocol and regular clinical supervision provided by the therapy’s originator. This hopes to ensure that memory reactivation occurs prior to reconsolidation, promote fidelity, and that the therapists, who will have prior training in other therapy modalities, avoid using these during sessions. The Rewind manual also attempts to improve tolerability and limit the distress and re-traumatisation that some participants experience with exposure work, by pausing when physiological arousal is reached and encouraging the participant to view their index trauma in the third person (Adams & Allan, 2018).

Other limitations of our work include those common to many empirical assessments of psychological therapy, such as it being almost impossible to conduct double-blind trials. All of the outcome assessments will, however, be conducted blinded to intervention group and participants will be asked not to disclose their treatment allocation.

To conclude, this study will be the first RCT to assess The Rewind technique, a modality of therapy currently already being utilised to treat PTSD. The study will generate significant new knowledge, given its diversity of outcomes, quality of outcome measurements, trial methodology and breadth of scope. Its challenges will include participant recruitment and retention, as well as ensuring strong adherence to the intervention protocol.

## Data Availability

As a protocol there are no publicly available data for this work.
